# Education Modifies Genetic and Environmental Influences on BMI

**DOI:** 10.1371/journal.pone.0016290

**Published:** 2011-01-19

**Authors:** Wendy Johnson, Kirsten Ohm Kyvik, Axel Skytthe, Ian J. Deary, Thorkild I. A. Sørensen

**Affiliations:** 1 Centre for Cognitive Ageing and Cognitive Epidemiology, Department of Psychology, University of Edinburgh, Edinburgh, United Kingdom; 2 Department of Psychology, University of Minnesota, Twin Cities, Minnesota, United States of America; 3 Institute of Regional Health Services Research, University of Southern Denmark, Odense, Denmark; 4 Danish Twin Registry, Epidemiology, Institute of Public Health, University of Southern Denmark, Odense, Denmark; 5 Institute of Preventive Medicine, Copenhagen University Hospital, Copenhagen, Denmark; Africa Centre for Health and Population Studies, University of KwaZulu Natal, South Africa

## Abstract

Obesity is more common among the less educated, suggesting education-related environmental triggers. Such triggers may act differently dependent on genetic and environmental predisposition to obesity. In a Danish Twin Registry survey, 21,522 twins of same-sex pairs provided zygosity, height, weight, and education data. Body mass index (BMI = kg weight/ m height^2^) was used to measure degree of obesity. We used quantitative genetic modeling to examine how genetic and shared and nonshared environmental variance in BMI differed by level of education and to estimate how genetic and shared and nonshared environmental correlations between education and BMI differed by level of education, analyzing women and men separately. Correlations between education and BMI were −.13 in women, −.15 in men. High BMI's were less frequent among well-educated participants, generating less variance. In women, this was due to restriction of all forms of variance, overall by a factor of about 2. In men, genetic variance did not vary with education, but results for shared and nonshared environmental variance were similar to those for women. The contributions of the shared environment to the correlations between education and BMI were substantial among the well-educated, suggesting importance of familial environmental influences common to high education and lower BMI. Family influence was particularly important in linking high education and lower levels of obesity.

## Introduction

Twin and adoption studies have demonstrated conclusively that body weight is under genetic influence [1] [2] [3]; yet genomewide association studies reveal that the phenotypic variance associated with any one genetic variant is very small [4]. Obesity must occur through genetic expression, probably of a very large number of genes, and some expression patterns may not even involve genetic differences among individuals. The same twin and adoption studies that demonstrated genetic influence have also shown that there are important environmental influences on body weight, and the ongoing obesity epidemic must be due to changes in some environmental exposures. Obesity is thus a multi-factorial abnormality that has a genetic foundation, but is more likely to be manifested in some environmental circumstances than others.

Many specific factors involving environment are also associated with obesity, including socioeconomic status (SES), education [5], stress [6], and social clustering [7]. In developed societies, obesity is more common among those with fewer economic resources and less education [5], making it part of the well-established SES-health gradient [8], the tendency for those with more economic resources and education to have better physical health. This gradient is continuous, with even those at the highest levels of SES having better outcomes related to health than those just below them. Though of course SES encompasses other dimensions, particularly economic resources and their associates, we focused in this study on education because it better reflects a life-long stable characteristic. Possible reasons for the gradient are not mutually exclusive. The better-educated tend to live in better environments [9]. There may be some form of genetic physiological robustness that influences both the maintenance of appropriate weight and the pursuit of education [10]. Better-educated people may know more about how to take care of themselves [11], and obesity may impede acquisition of a good education [12] [13].

Genetic influences on obesity may involve not merely metabolic and physiological characteristics, but also psychological characteristics. Some of these psychological characteristics could overlap with those involved in educational attainment. For example, a psychological characteristic such as self-discipline may be used to study hard in order to acquire more and better education and also to restrain eating and maintain an exercise program. There may also be familial and cultural influences that contribute both to educational attainment and maintenance of appropriate weight [5] [7] [14]. Moreover, every behavior involving choice shows genetic as well as environmental influences [15]. When the level of one genetically influenced trait contributes to choices involved in some environmentally influenced outcome, the genetic influences on the trait will also show up as genetic influences on the environmental outcome. This is known as active gene-environment correlation, social selection, or niche-picking [16]. In addition to possible genetic correlation, gene-environment interaction is involved in obesity [4]. Gene-environment interaction occurs when genetic differences make people respond differently to environmental circumstances. Due to genetic differences, people's weights are differentially sensitive to over- and underfeeding, as well as to consumption of different kinds of foods, to physical activity, and to both psychological and physiological stress [17] [18] [19].

Two previous studies have suggested that such gene-environment correlation and interaction processes may be involved in the greater frequency of obesity and/or high body mass index (BMI; kg weight/m height^2^) among the less educated. In one United States sample [20], [21], genetic as well as total variance in both BMI and physical health was greater among those with less education. The finding with respect to physical health was largely replicated in a much larger Danish sample [22]. This is important because the United States and Denmark differ considerably in SES disparities, access to education, and allocation of income within their populations it has been suggested that such factors involving social structure may be among the root causes of the physical health/obesity gradient. The purpose of this study was to use this same sample to explore the degree to which similar processes might be involved in the association between education and degree of obesity as indicated by BMI.

## Methods

### Source Data

The Danish Twin Registry was established in 1954. The oldest in the world, it includes twin births from 1870–2004. More than 75,000 twin pairs have been registered to date. For this study, we made use of data from a questionnaire mailed in 2002 to 46,333 Registry participants born from 1931 to 1982. Participants thus ranged in age at time of response from 20 to 71, with means of 43 (SD = 14) for females and 44 (SD = 14) for males. The Scientific Ethical Committee of the Danish counties of Funen and Vejle approved the questionnaire, and participants gave permission for use of their data through their survey responses. Health and education were not its primary focus, but it included health-related questions including self-reported height and weight as well as highest education attained for twin and spouse. In total, 34,944 individuals (75.4%) responded by completing the questionnaire. Standard questions on physical likeness and mistaken identity were used to determine twin zygosity [23]. This form of zygosity assessment is valid, with a misclassification rate of only 4% [24]. Of the 34,944 respondents, 5,024 were female monozygotic (MZ) twins, 6,785 were female same-sex dizygotic (DZ), 6,652 were female opposite-sex, 3,976 were male MZ, 6,092 were male same-sex DZ, and 5,265 were male opposite-sex twins, with the remainder missing zygosity information. We used the 21,522 same-sex twins with zygosity information and usable data for either self-reported height and weight or education (365 were missing both these variables). There were approximately 500 reports of heights less than 140 cm., which generated impossibly large BMI's. We treated all BMI's in excess of 70 as missing.

Table 1 provides descriptive statistics for these variables. All sex differences were highly statistically significant so we treated women and men separately throughout. The participants were relatively uniformly distributed throughout the 1931 through 1982 birth cohorts, with women born on average in 1958 and men born on average in 1957. Average level of education corresponded to completion of secondary school examination, or some combination of secondary education without examination and supplementary vocational training. Women had completed slightly more education than had men, with a difference of .18 SD. Average BMI was 24.46; as shown in the table, it was greater for men than for women, with a difference of .42 SD. At the same time, variance in BMI for women was much greater than that for men (women∶men variance ratio = 1.57). Both BMI and education showed associations with participant year of birth (those with earlier years of birth were less educated and had higher BMI's), effects that were likely cohort-related for education and age-related for BMI. Birth-year effects can also act inappropriately to inflate the similarity between co-twins because twins are the same age [25]. To remove these effects, we regressed BMI and education on age and age-squared separately by gender, and analysed the residuals. BMI was positively skewed so we log-transformed it prior to further analysis, making it approximately normal in distribution.

**Table 1:** Descriptive statistics for study variables.

Variable	Women (n = 11,607)		Men (n = 9,915)		Standardized
	Mean	Std. Dev.	Mean	Std. Dev.	Mean Difference
Year Born	1958.92	13.77	1957.65	13.76	.09
Education	6.90	3.31	6.29	3.28	.18
Body Mass Index	23.61	4.32	25.29	3.44	−.42

Note: Education was scaled so that completion of Grade 7 with no further training was scored 0 and completion of education beyond a university degree was scored 12. Intermediate scores reflected both greater formal schooling and vocational training.

### Twin Models for Examining Gene-Environment Correlation and Interaction

To understand how we used the twin sample to estimate gene-environment correlation and interaction, it helps to outline the process through which the model we used was derived. We relied on the standard assumption of the quantitative genetic model that variance in BMI could be attributed to three sources, often called components: additive genetic influences (A), shared environmental influences that made twins in the same pair more similar but differentiated among twin pairs (C), and non-shared environmental influences including measurement error that made all twins different from each other regardless of zygosity or family membership (E). Under this model, because MZ twins share all their genes and DZ twins share on average 50% of their segregating genes, a higher correlation in BMI between MZ twins than DZ twins indicates additive genetic influences. If the DZ correlation in BMI is greater than one-half the MZ correlation, shared environmental influences on their similarity are indicated. MZ Twin correlations less than 1.0 indicate non-shared environmental influences [16].

Using basic matrix algebra, this univariate model can be extended to estimate the genetic and environmental contributions to the covariance between education and BMI. The extended model includes estimates of A, C, and E influences on education that also contribute to BMI, thus creating their covariance, and A, C, and E influences that contribute to BMI alone. The genetic correlation (r_A_) is the standardized genetic covariance. Like ordinary phenotypic correlations, it varies from 1.0 to −1.0, but it indexes the extent to which genetic influences on education and BMI covary. When there is genetic correlation between a trait and a circumstance considered environmental, the genetic correlation is often referred to as gene-environment correlation. Education is an example of such a circumstance. Many think of it as environmental, but it shows substantial genetic influences as well (e.g., [26]).

When genetic correlation is high, similar genetic influences contribute to two distinguishable characteristics. This can happen in several different ways. It can happen because individual genes are pleiotropic: they contribute directly to both characteristics, possibly by different mechanisms. For example, there could be genes that contribute to self-discipline that, in turn, results both in study effort and restraint in eating, or there could be genes that simply have effects on both intelligence and body weight through different biological pathways. High genetic correlation can also happen because two different genes that are closely linked in the genome (and so generally transmitted together) contribute to each characteristic. And it can happen because one genetically influenced trait contributes directly to the other. For example, there could be genes that contribute to educational failure, which then causes overeating and lack of exercise due to lack of occupational opportunity and associated depression, leading to obesity. The analogous shared (r_C_) and non-shared environmental (r_E_) correlations are estimated in the model and can be interpreted in similar ways.

**Figure 1:**
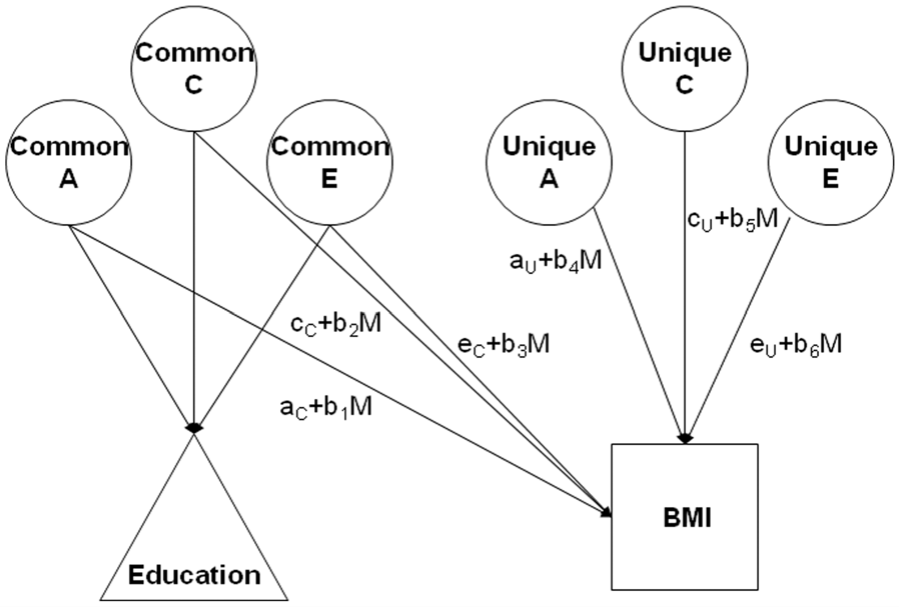
Model of moderation of genetic and environmental influences on BMI as moderated by education. A refers to variance components attributable to genetic influences, C to variance components attributable to shared environmental influences, and E to variance components attributable to nonshared environmental influences. Education is represented as a triangle because it is conceptualized as an environmental moderating variable with respect to BMI. Variance in BMI may be influenced by any or all six of the paths shown, and the extents of these influences may themselves vary linearly with Education, noted by M for moderating variable in the paths above. The genetic and environmental variance components influencing Education are also shown in the figure, but not labeled. The c and u subscripts refer to influences common to Education and BMI and unique to BMI alone.

The model we used had one additional extension. The models described so far provide estimates of A, C, and E influences applicable to the population at large assuming there are no interactions or correlations among the sources of influence, and that the influences are constant throughout the population. We used a model that allowed these assumptions to be relaxed so that the possibilities that the variance components differed in different parts of the population could be examined. Differences in the genetic variance component of BMI with differences in level of education would be an example of G×E interaction, arising from differential genetic sensitivity of BMI to the environmental circumstances associated with level of education. Additionally, the genetic and environmental correlations between education and BMI might vary with level of education. For example, the r_A_ between education and BMI could be greater at higher levels of education (or vice versa). This would indicate that genetic differences were involved in either the ability to use education to move away from undesirable environments that act to increase BMI or the ability to use education to minimize the effects of such environments, or both. Under this model, instead of being fixed constants, the genetic and environmental variance components of the trait in question, here BMI, are considered to be linear functions of an environmental moderating variable, here education. Moderating effects are possible for genetic and shared and nonshared environmental variance components both common to education and BMI and unique to BMI. Figure 1 is a diagram of the model. In this figure, the parameters indicating moderation that were of particular interest in this study are b_1_ through b_6_ that apply to genetic and environmental influences on M, referring to Education, the moderating variable. When these coefficients were significant, genetic and environmental influences on BMI varied with level of Education.

### Statistical Analysis

**Figure 2:**
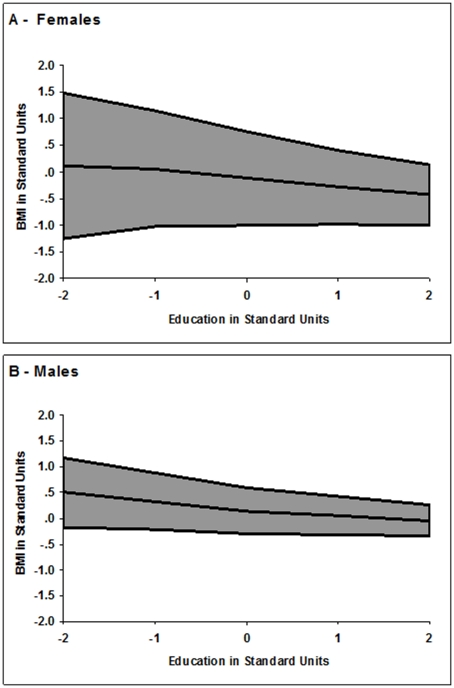
Total variation around mean levels of BMI, as functions of educational attainment. The x-axes represent educational attainment in standard deviation units, birth year effects removed. The y-axes represent variation around mean levels of BMI in standard units, birth year effects removed. Males and females are shown on the same scale so that variances are comparable.

Purcell [27] has articulated several genetic-environmental moderator models, implemented them in Mx software [28], and made them freely available on his website (http://pngu.mgh.harvard.edu/~purcell/gxe/). We used the ‘GxE in the presence of rGE’ model, which operationalizes the model shown in Figure 1. The Mx program uses maximum likelihood estimation so that all twin data can be included, regardless of co-twin data availability. This model enabled us to measure: 1) the extent to which variance in BMI could be attributed to genetic and environmental influences (the various a, c, and e parameters in Figure 1); 2) differences in BMI variance with level of education (the b coefficients in Figure 1 as noted above); and 3) the extent to which the same influences contributed to both education and BMI, as reflected by correlations of genetic and shared and non-shared environmental influences on education and BMI (formed from the a, c, and e parameters with subscripts c in Figure 1).

Of course people's education did not vary at any specific point in its range, but these correlations could still vary with level of education. This is because there was considerable genetic and environmental variance in the pathways through which people attained any given level of education, and it was possible that some of this variance overlapped with that in BMI. Importantly, because we were interested in differences in overall variance with education as well as differences in means, we estimated absolute genetic and environmental variance components, and only converted them to the more commonly expressed proportions of total variance secondarily. In some situations our model can produce spurious or uninterpretable results [29], but those situations did not apply here: the positively skewed BMI variable was reasonably normally distributed when log-transformed, moderation was on variance unique to BMI rather than variance shared with education, and variance in BMI was not dependent on level of BMI, leaving the results we observed robust to transformation of scale.

Because of the complexity of our model, we allowed parsimony to dictate the results presented. We tested the significance of the terms indicating moderating effects of education on BMI (the b coefficients in Figure 1) and dropped them when we could do so without significant change in model −2*log likelihood. We evaluated the appropriateness of this using the information theoretic fit statistics Akaike's Information Criterion (AIC) [30] and the Bayesian Information Criterion (BIC) [31]. We dropped non-significant moderating terms not to deny the potential existence of smaller moderating effects that happened not to be significant in this particular sample but to focus attention on the effects of education that were most important in these data. Given our large sample, the moderating effects we dropped were not of substantive importance.

## Results

Table 2 presents the models we tested and the fit statistics associated with them. The best-fitting models for women and men were very similar. For women, all the parameters indicating moderation by Education of the genetic and environmental influences on both BMI and Education could be constrained to 0 without loss of fit. Fixing any of the parameters indicating moderation by Education on the genetic and environmental influences unique to BMI, however, caused deterioration in model fit. The situation with respect to the genetic and environmental influences on both BMI and Education was the same in men. In addition, there was no evidence that Education moderated the genetic influences unique to BMI. Fixing the parameters indicating moderation by Education on the shared and nonshared environmental influences unique to BMI, however, caused deterioration in model fit.

**Table 2:** Fit statistics from the models of variance components of education and body mass index allowing for gene-environment and correlation.

Model	−2*LL	df	χ^2^	Δdf	*p*	AIC	BIC
Body Mass Index - Females							
All parameters free	41141.7	23982	—	—	—	41163.7	41224.2
Fix common A, C, and E moderation paths*	41147.5	23985	5.8	3	ns	41163.5	41207.5
Fix all moderation paths	41257.2	23988	109.7	3	<.001	41267.2	41294.7
Body Mass Index - Males							
All parameters free	27853.3	17980	—	—	—	27875.3	27934.8
Fix common A, C, and E and unique A moderation paths*	27860.1	17984	6.8	4	ns	27874.1	27912.0
Fix all moderation paths	27936.1	17986	76.0	2	<.001	27946.1	27973.2

Note: A refers to genetic influences, C to shared environmental influences, and E to nonshared environmental influences.

There are possible common and unique moderation paths for each of A, C, and E. Best-fitting models are indicated by *. Fixed moderation paths were constrained to 0, which means that those sources of influence were present but did not vary across the levels of the moderators. AIC is Akaike Information Criterion. BIC is Bayesian Information Criterion. Because of the large sample size and number of statistical tests performed, we set the significance level for the chi-squared tests at .01.

Figure 2 shows how mean levels and variance in BMI differed with level of education, separately for women and men. BMI was standardized to z-scores on the full sample to make gender differences in either means or overall variances clearly visible. The thicknesses of the bands along the y-axis show the total BMI variance at different levels of education (x-axis), and the overall levels of the bands show the effects on the mean level. There was much more variation in BMI among people with low levels of education, and much more variance in BMI in women than men. Still, for both genders, variance in BMI at 2 standard deviations above the mean level of education was about half that at 2 standard deviations below the mean. One explanation for this is that lack of education was a marker of environmental conditions that trigger greater expression of vulnerabilities to high BMI, but there were large individual differences in these vulnerabilities. If education in fact drove this expression, some people, but not others, may have used higher education either to make better environmental circumstances or to control BMI or both, but lower levels of education apparently did not tend to make this possible. In this event, individual differences both in vulnerability to obesity and in use of higher education, led to reduced variance in BMI among those with higher education.

### Moderating Effects

**Figure 3:**
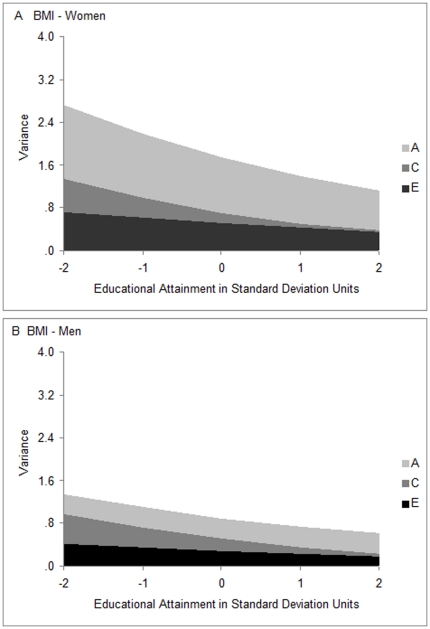
Variance components of BMI as functions of educational attainment. The x-axes represent educational attainment in standard deviation units. The y-axes represent variance in BMI in standard units. Males and females are shown on the same scale so that variances are comparable, and birth year effects have been removed. Variance components attributable to genetic influences (labeled A as is common) are shown in light gray. Variance components attributable to shared environmental influences (labeled C), familial and local community influences that make members of twin pairs similar, are shown in dark gray. Variance components attributable to nonshared environmental influences(labeled E) that produce differences in BMI in members of twin pairs, are shown in black.

Figure 3 shows the results of separating the variance by genetic and environmental source, again separately for women and men, and Table 3 presents them in tabular form together with confidence intervals. In addition to the variance components for BMI, the table shows the constant variance components for education. Additive genetic variance in BMI is shown with light gray bands in the figure, shared environmental variance in BMI is shown with dark gray bands, and non-shared environmental variance in BMI is shown with black bands. There was less additive genetic variance (light gray bands) at higher levels of education in women (.76 at 2 standard deviations above mean education vs. 1.36 at 2 standard deviations below; Table 3), but additive genetic variance was constant across the range of education in men. There was also less shared environmental variance (dark gray bands) at higher levels of education, this time in both genders. It was substantial at low levels of education and essentially absent at high levels of education. There was less non-shared environmental variance (black bands) at higher levels of education in both genders as well, but the moderating effect of education was smaller. Thus, the novel results here were that BMI variation was moderated by level of education, and the principal target of the moderation was shared environmental variation, the expression of which was restricted markedly at higher levels of education. In fact, because education restricted environmental variance more than genetic variance, heritability, or the proportion of total variance attributable to genetic influences, was higher at high levels of education in both genders (see Table 3).

**Table 3:** Estimated variance components and proportions of variance in BMI in women and men and genetic and environmental correlations with education, at 3 levels of education.

	Education		BMI Moderated by Education, At Level of Education	
		−2 sd	0 sd	2 sd
Variance components		Women		
Genetic	.28	1.36	1.04	.76
	(.22,.34)	(1.12,1.62)	(.78,1.29)	(.41,.1.15)
Shared environmental	.35	.63	.19	.02
	(.29,.41)	(.44,.83)	(.07,.44)	(.00,.17)
Nonshared environmental	.29	.72	.52	.36
	(.27,.31)	(.63,.82)	(.43,.62)	(.24,.50)
Proportions of variance				
Genetic	.30	.51	.59	.66
	(.25,.35)	(.28,.74)	(.45,.69)	(.44,.88)
Shared environmental	.38	.23	.11	.02
	(.33,.43)	(.08,.39)	(.02,.25)	(.00,.12)
Nonshared environmental	.32	.26	.30	.32
	(.30,.34)	(.04,.39)	(.23,.38)	(.08,.54)
Correlations w/Moderator				
Genetic	N/A	−.08	−.09	−.11
		(−.16,.00)	(−.17,.00)	(−.21,.00)
Shared environmental	N/A	−.18	−.32	−.96
		(−.56,.00)	(−.68,−.10)	(−1.0,−.32)
Nonshared environmental	N/A	−.01	−.02	−.02
		(−.05,.02)	(−.05,.02)	(−.08,.02)
Variance components		Men		
Genetic	.47	.38	.38	.38
	(.40,.55)	(.35,.43)	(.35,.43)	(.35,.43)
Shared environmental	.26	.56	.23	.05
	(.19,.33)	(.36,.76)	(.17,.30)	(.00,.36)
Nonshared environmental	.27	.41	.29	.19
	(.24,.28)	(.32,.50)	(.27,.31)	(.11,.27)
Proportions of variance				
Genetic	.47	.28	.42	.61
	(.40,.55)	(.25,.31)	(.39,.47)	(.00,.77)
Shared environmental	.26	.41	.25	.08
	(.19,.33)	(.24,.58)	(.20,.32)	(.00,.38)
Nonshared environmental	.27	.31	.33	.31
	(.24,.28)	(.23,.39)	(.31,.35)	(.24,.38)
Correlations w/Moderator				
Genetic	N/A	−.17	−.17	−.17
		(−.25,−.07)	(−.25,−.07)	(−.25,−.07)
Shared environmental	N/A	−.14	−.22	−.49
		(−.58,.23)	(−.59,.00)	(−1.00,.10)
Nonshared environmental	N/A	.02	.02	.02
		(−.04,.09)	(−.04,.09)	(−.04,.09)

Note: The variance components are raw; they do not sum to 1.00. The proportions of variance sum to 1.00. 95% confidence intervals are given in parentheses. N/A is not applicable.

### Correlated Genetic and Environmental Effects

**Figure 4:**
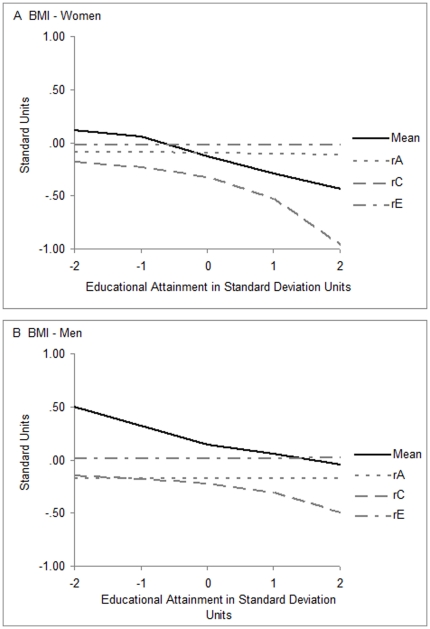
Mean BMI, birth year effects removed, and its correlations with educational attainment, as functions of educational attainment. The x-axes represent educational attainment in standard deviation units, birth year effects removed. The y-axes represent standard deviation units for the mean effects and standardized units for the correlations. The solid black lines, showing mean levels, indicate the BMI with education. Short-dashed gray lines indicate genetic correlations (rA), or the degrees to which the same genetic influences contributed to both education and BMI. Long-dashed gray lines indicate shared environmental correlations (rC), and uneven-dashed lines nonshared environmental correlations (rE).

The overall observed correlations between BMI and education were −.13 in women and −.15 in men. Figure 4 shows how the mean levels and the contribution of the genetic and shared and non-shared environment to the correlations between BMI and education varied with level of education, with tabular data given in Table 3 together with confidence intervals. The black lines in panels A and B of the figure show the mean levels of BMI (y axis) in standard units at different levels of education (x axis). As the correlations indicated, BMI was greater in people with less education. The three dashed gray lines in Figure 4 represent the correlations between the relevant sources of influences on education and the same sources of influences on BMI (y axis) in relation to level of education (x axis). The short-dashed gray lines represent the extent to which the same genetic influences accounted for variance in both BMI and education. The lines also show how these correlations differed with level of education. The long-dashed gray lines provide the same information for non-shared environmental influences. In both genders, both genetic and nonshared environmental correlations were low and nonsignificant across the range of education (Table 3; −.17 for the genetic correlation for men; otherwise the confidence intervals included 0).

In sharp contrast, the uneven-dashed gray lines in Figure 4, which show the shared environmental correlations, indicate large differences with level of education in both genders. The correlations were small (negative) when level of education was low, but became increasingly strongly negative at higher levels of education. The fact that the correlations were negative indicated that familial and local community influences making twins similar and contributing to greater education helped to control BMI, and the increasing strength of the correlations with level of education indicated that this was true to a greater degree when level of education was high than when it was low. Genetic and environmental correlations are not generally measured with much precision [32], so the differences in women and men, though appearing substantial, were not significant.

## Discussion

In this study, we used a large population-representative twin sample from Denmark to explore how genetic and environmental interaction and correlation processes might be involved in the association of education with both levels of and variance in obesity as measured by BMI. As has been noted by many others (e.g., 5), higher levels of education were associated with lower BMI in our sample. From slightly different perspectives, many have suggested that this association exists because environmental conditions associated with low levels of education limit access to and knowledge of nutritious food choices and safe means to exercise [9] [14], bring on metabolic dysregulation [33], and trigger higher caloric consumption [6]. We investigated these ideas by measuring how education moderated the variance in BMI attributable to genetic and shared and non-shared environmental influences and the associated genetic and shared and nonshared environmental contributions to the correlations.

Variance in BMI was lower among the more educated primarily because the highest BMI's were very rare, which also meant lower mean levels of BMI among the more educated. One explanation for this is that lack of education was a marker of environmental conditions that triggered greater expression of vulnerabilities to high BMI. Because there was more variance among the less educated, there also appeared to be substantial individual differences in these vulnerabilities. The fact that variance attributable to environmental influences was responsive to level of education indicated that familial/cultural heterogeneity as well as circumstances unique to each individual were likely involved in the expression of the individual differences. Genetic variance also showed evidence of these vulnerabilities to environmental conditions associated with lack of education in women, though not in men. This is a form of interaction of influences. In this case, the shared environmental influences showed particularly strong interaction, but genetic influences also interacted with the environments created by level of education in women.

In both genders, variance attributable to shared environmental influences was particularly responsive to level of education. In women, it increased from .02, or 2% of total variance, at 2 standard deviations above the mean level of education to .63, or 23% of total variance, at 2 standard deviations below the mean (Table 3). In men, it increased from .05, or 8% of total variance, to .56, or 41% of total variance, over the same range. Because this increase was sharper than the increases in variance attributable to genetic and non-shared environmental influences, heritability of BMI was lower among those with low levels of education than among those with high levels. Thus, in women, it decreased from 66% to 51% over the 4-standard deviation range; in men, it decreased from 61% to 28% (Table 3).

The patterns of genetic and shared and non-shared environmental correlations between education and BMI provided important clues to the social systems involved in the association between education and BMI in this sample. The very low and stable genetic and non-shared environmental correlations in both women and men indicated that there was little reason to expect population stratification with respect to education of whatever genes are involved in BMI. The confidence intervals for these correlations included 0, suggesting they were not significant. Such correlations can never be measured with much precision. They were, however, products of the highly significant pattern of less shared environmental variance with more education, making the pattern they suggest of importance. That is, at least for genetic and non-shared environmental reasons, people with low levels of education were as likely to have high BMI as those with high levels of education. Thus, we would not expect to find much in the way of different frequencies of genes associated with high BMI in groups of people with different levels of education. The substantial inverse shared environmental correlations at high levels of education, however, suggested that cultural/familial influences on high educational attainment acted relatively uniformly and effectively to reduce BMI by restricting variance. At the same time, cultural/familial influences on lower levels of educational attainment were much less effective in controlling BMI, and there was thus much greater heterogeneity in the shared environmental influences on BMI. This means that we would expect to find much greater homogeneity of and - from a health-related perspective - better cultural/familial influences on food, exercise, and stress management choices among those with high levels of education than among those with low levels of education. Many studies from very different perspectives have indicated that this is the case. Because our sample consisted of adult twins living independently, it is likely that the habits and metabolic responses associated with the shared environmental influences were formed early in life when the twins were living together in childhood, or perhaps even prenatally [9] [34].

We noted both higher average BMI in men and substantially greater variance in BMI in women. A greater and more variable tendency to underreport body weight by women may have contributed. Men have on average greater lean body mass and smaller fat body mass than women , and fat body mass shows greater variability than lean body mass. There are many possible reasons for greater variance in BMI in women. It could be attributed primarily to genetic and non-shared environmental sources, but this does not mean that it cannot be attributed to systematic cultural/familial gender differences in messages about nutrition, exercise, body image, etc, as well as response to pregnancy. It is possible that such sources could be part of the environmental conditions that may trigger greater expression of genetic and environmental vulnerabilities to obesity.

Our results in this study showed both consistencies with and differences from those of the similar smaller study using a sample from the United States [20]. In that study, the moderating variable was income. Socio-economic status (SES) is usually indicated by some composite of education, occupation and income, so both studies touched on the involvement of BMI in the SES-health gradient, but they did so from slightly different perspectives. The US-based sample was too small to examine effects separately in women and men, but, consistent with the results of this study in women, the US-based study showed greater variance in BMI at lower levels of income, and the variance could be characterized as being due to genetic differences. The overall pattern of restricted variance with higher SES is important in understanding the association between SES and BMI [5]. Moreover, genetic variance in physical health has shown a similar association with education in this Danish sample [21] and to income in the US-based sample [31]. Taking these studies together (important in analyses of interactive effects of all kinds), it appears that the environment associated with low SES is also associated with poorer health primarily because it increases expression of genetic vulnerabilities to health problems - including obesity - that are carried to varying extents by all humans. The similarity of the genetic response in Danish women to that in the United States was particularly noteworthy, as the two countries differ considerably in disparity in SES within their populations, as well as in allocation of medical care. In the US-based study of moderating effects of income on BMI, income did not moderate variance in BMI attributable to either shared or non-shared environmental influences, unlike the results of this study. There are many possible reasons for this, including relative lack of power to detect effects in the US-based sample and many possible differences in national culture in the two countries. Another likely reason, however, is the difference in the moderating variable as a reflection of SES. Education tends to show much stronger shared environmental influences than income in most samples, as parents work to equalize educational opportunities for their offspring [34]. Income tends to show stronger genetic influences.

Despite its well-characterized, large population-representative nationwide sample, this study had limitations. The heights and weights used to calculate BMI were obtained by self-report. Self-reports of BMI have tended to be biased. Specifically, under-reporting of weight and over-reporting of height tends to lead to obesity prevalence rates that are too low [35], [36], and this tendency is generally greater in those with higher BMIs [37] and those with lower income. Women tend to report more accurately than men, and younger people more accurately than older people [36], [37]. Overall, these biases would act to reduce the apparent magnitudes of the effects we observed, meaning that it is likely that actual associations with education were stronger than those we report.

BMI itself is a rather crude measure of obesity, as it does not recognize individual differences in body composition with regard to fat and lean mass. The sample had a wide range of ages, and age was associated with BMI and education in the data. We accounted for the direct effects of age in our models, but there may have been indirect or interactive effects for which we did not account. Though we treated it as measured on an interval scale, education does not naturally lend itself to this. Obviously, our results apply to twins in the age and geographical group studied here, though, as noted, other data from the United States have shown some similar effects. Finally and importantly, our characterization of influences as additive genetic includes pre- and perinatal environmental effects, possibly resulting in epigenetic differences that also operate to make MZ twins more similar than DZ twins. Similarly, our characterizations of influences as shared and non-shared environmental include these kinds of effects that also operate to make twin pairs similar regardless of zygosity (shared) and different from each other (non-shared).

In conclusion, this study indicated that variance in BMI in general and variance attributable to shared environmental influences on BMI in particular was greater among people with low levels of education than among those with higher levels of education. This was primarily because it was more common for people with less education to develop the very high BMI's associated with obesity. This is compatible with theoretical models in which the cultural/familial influences associated with high educational attainment also regulate body weight. In a complex model like this, our specific results could reflect a fine balance among many different psychosocial forces. Future research should therefore seek not only to determine how replicable these findings might be, but to identify the specific metabolic pathways and environmental circumstances that bring these forces together in particular ways in varying environmental and personal circumstances.
